# Prognostic significance of PD-L1-positive cancer-associated fibroblasts in patients with triple-negative breast cancer

**DOI:** 10.1186/s12885-021-07970-x

**Published:** 2021-03-06

**Authors:** Katsuhiro Yoshikawa, Mitsuaki Ishida, Hirotsugu Yanai, Koji Tsuta, Mitsugu Sekimoto, Tomoharu Sugie

**Affiliations:** 1 Department of Pathology and Clinical Laboratory, Kansai Medical University, 2-5-1, Shinmachi, Osaka 573-1010 Hirakata City, Japan; 2 Department of Surgery, Kansai Medical University, 2-5-1, Shinmachi, Osaka Hirakata City, Japan

**Keywords:** Triple-negative breast cancer, Programmed death ligand 1, Cancer-associated fibroblasts, Overall survival

## Abstract

**Background:**

Cancer-associated fibroblasts (CAFs) are some of the most abundant components of the tumour microenvironment. A recent study suggested that in some cancers, CAFs express programmed death ligand 1 (PD-L1), which can act as a prognostic marker. The aim of this study was to investigate the clinicopathological significance of CAF PD-L1 expression in patients with triple-negative breast cancer (TNBC) and to identify the most suitable primary antibody for immunostaining for CAF PD-L1.

**Methods:**

Immunohistochemical staining (primary antibodies of 73–10, SP142, and E1L3N) and tissue microarrays were used to analyse the expression profiles of PD-L1 in CAF in 61 patients with TNBC who underwent surgery. Overall survival (OS) was compared based on CAF PD-L1 expression, and the risk factors for OS were analysed. The relationship between clinicopathological parameters and survival was also examined.

**Results:**

Thirty-four (55.7%) patients were positive for CAF PD-L1 (73–10) expression. Compared with CAF PD-L1 negativity, there was a significant correlation between CAF PD-L1 positivity and better OS (*p* = 0.029). CAF PD-L1 expression, evaluated using SP-142 or E1L3N, did not correlate with OS. CAF PD-L1-positivity (73–10) correlated significantly with better prognosis in multivariate analyses (hazard ratio: 0.198; 95% confidence interval: 0.044–0.891; *p* = 0.035).

**Conclusions:**

CAF PD-L1 expression is a novel marker for a better prognosis of patients with TNBC, and the 73–10 assay may be suitable for immunostaining CAF PD-L1.

## Background

Breast cancers usually influence the immune system; however, the level of immune infiltration reportedly differs among breast cancer molecular subtypes [1]. Triple-negative breast cancer (TNBC), characterised by the lack of oestrogen and progesterone receptors and human epidermal growth factor receptor 2 (HER2) expression, is a more aggressive breast cancer than other subtypes that correlate with poor prognosis [2, 3]. Most patients with TNBC are categorised as having a robust tumour lymphocytic infiltrate compared to other subtypes. Therefore, TNBC is considered the most immunogenic subtype [4]. Recently, the expression of programmed death ligand 1 (PD-L1) in tumour cells and/or immune cells in breast cancer tissues has received much attention because of its effectiveness in anti-PD-L1/PD1 targeted therapy [5, 6]. It has been reported that PD-L1 expression in tumour cells was associated with higher histological grade, hormone receptor-negative phenotype, poor prognostic outcome, and lymph node status in breast cancer [7–9]. Moreover, previous studies revealed that PD-L1 expression in both tumour and immune cells was the highest in TNBC compared to other subtypes [10–12].

Cancer-associated fibroblasts (CAFs) are considered to produce a pro-tumourigenic microenvironment and are some of its dominant components [13]. CAFs have been known to play important roles in cancer growth, invasion, metastasis, and therapeutic resistance through the secretion of various soluble factors, including chemokines, growth factors, and exosomes in some types of carcinomas, including breast cancer [4, 13–17]. Although PD-L1 expression has been demonstrated in subsets of tumour-infiltrating lymphocytes and macrophages, its expression in CAFs has not yet been analysed in detail. Recently, PD-L1 expression in CAFs of non-small cell lung carcinoma tissues has been demonstrated to correlate with good patient prognosis [18]. However, PD-L1 expression in CAFs has never been analysed in breast cancer tissues, and its prognostic significance remains to be clarified. Thus, the aim of this study was to determine PD-L1 expression in CAFs of TNBC tissues and its prognostic significance in patients with TNBC.

## Methods

### Patient selection

We enrolled 165 consecutive patients with TNBC who underwent surgical resection from January 2006 to December 2018 at the Department of Surgery of the Kansai Medical University Hospital. Patients who received neoadjuvant chemotherapy were excluded from the study because this chemotherapy may influence PD-L1 expression and may also have an impact on the patient prognosis. Patients who were diagnosed with invasive carcinoma of no special type according to the recent World Health Organization Classification of Breast Tumours [19] were selected. Patients with special types of invasive carcinoma were excluded from the study, because each special type of carcinoma has unique clinicopathological features; hence, 61 patients with TNBC comprised this study cohort. The present cohort was fundamentally the same as that of our previous study [20]. In the previous study, we analysed the relationship between adipophilin expression, a lipid droplet-associated protein, and the clinicopathological features of TNBC patients. The content of this study did not overlap with that of our previous one [20].

This study was conducted in accordance with the Declaration of Helsinki, and the study protocol was approved by the Institutional Review Board of the Kansai Medical University Hospital (Approval #2019041).

### Histopathological analysis

Histopathological features were independently evaluated by more than two experienced pathologists. We used the TNM Classification of Malignant Tumours, Eighth Edition. Histopathological grading was based on the Nottingham histological grade [21]. The Ki-67 labelling index (LI) was considered high when ≥40% of the neoplastic cells were labelled [22]. Stromal tumour-infiltrating lymphocytes (TILs) were identified using haematoxylin and eosin staining, and were considered lymphocyte-predominant breast cancer (LPBC) at ≥60% and non-LPBC at less than 60%, according to the TIL Working Group recommendation [23, 24].

### Tissue microarray

The most morphologically representative carcinoma regions were selected on haematoxylin and eosin-stained slides, and three tissue cores (2 mm in diameter) were punched out from the paraffin-embedded blocks for each patient. These tissue cores were arrayed in a paraffin blocks.

### Immunohistochemistry

Immunohistochemical stainings were performed using autostainers (SP142 and E1L3N assays on Discovery ULTRA System; Roche Diagnostics, Basel, Switzerland; and 73–10 assay on Leica Bond-III; Leica Biosystems, Bannockburn, IL). Three different primary monoclonal antibodies were used to detect PD-L1: SP142 (Roche Diagnostics, Basel, Switzerland), E1L3N (Cell Signaling Technology, Danvers, MA, USA) and 73–10 (Leica Biosystems, Newcastle, UK). A minimum of two researchers independently evaluated the immunohistochemical staining results.

Spindle-shaped non-neoplastic cells in tumour stroma were morphologically recognized as CAFs, and membranous and/or cytoplasmic expression of PD-L1 in these cells was considered positive. PD-L1 expression scores of CAFs were determined based on the staining intensity and were classified into three levels (0, negative; + 1, weak; + 2, strong). As previously reported, CAF PD-L1 positivity was defined as the presence of CAFs with staining intensities of + 1 and + 2 in more than 1% of a section and positive immunoreactivity of ≥1 from the same patient [18]. In addition, PD-L1 expression in stromal TILs was defined as expression in more than 5% of TILs (TIL PD-L1-positive) [25, 26].

### Double immunofluorescence staining

For immunofluorescence analysis, a primary mouse monoclonal antibody against α-smooth muscle actin (α-SMA) (SPM332, Abcam, Cambridge, MA, USA) and primary rabbit monoclonal antibody against PD-L1 (73–10, ab228415, Abcam) were used. Subsequently, secondary antibodies of goat anti-rabbit immunoglobulin G (IgG) (Alexa Fluor® 488 [ab150081]) and goat anti-mouse IgG Alexa Fluor® 568 (ab175701) were used. The immunofluorescence stain was analysed using a fluorescence microscope (Olympus BX53F, Tokyo, Japan).

### Statistical analyses

SPSS Statistics 25.0 (IBM, Armonk, NY, USA) was used to perform the statistical analyses. Correlations between two groups were calculated using Fisher’s exact test for categorical variables and the Mann–Whitney *U* test for continuous variables. The rates of relapse-free survival (RFS) and overall survival (OS) were evaluated using Kaplan–Meier method. The Cox proportional hazards model was used to examine the relationship between clinicopathological parameters and survival. A multivariate analysis was performed using a step-wise method. A *p*-value of < 0.05 was considered to be significant.

## Results

### Clinicopathological features

This study comprised 61 female patients whose clinical characteristics are summarised in Table 1. The age of the patients ranged from 31 to 93 years (median 67 years). Based on biopsy results, all the patients had TNBC. Patients were staged as I (25 patients), IIA (23 patients), IIB (5 patients), IIIA (4 patients), IIIB (3 patients), and IIIC (1 patient). The median time of observation was 60 months (range, 11–163 months). Ten (16.4%) patients had a relapse (all experienced distant metastases). Nine (14.8%) patients died from the disease, and five (8.2%) patients died from other causes.

**Table 1:** Clinical characteristics of patients with triple-negative breast cancer

Factors	n	%
Total	61	
Age (years old)		
Median (range)	67 (31–93)	
Menopausal status		
Premenopausal	9	14.8
Postmenopausal	51	83.6
Unknown	1	1.6
Tumour size (mm)		
Median (range)	20 (2–55)	
Pathological stage		
I	25	41.0
IIA	23	37.7
IIB	5	8.2
IIIA	4	6.6
IIIB	3	4.9
IIIC	1	1.6
Lymph node status		
positive	14	23.0
negative	33	54.0
not tested	14	23.0
Lymphatic invasion		
positive	52	85.2
negative	9	14.8
Venous invasion		
positive	37	60.7
negative	24	39.3
Nottingham histological grade		
1	2	3.3
2	27	44.3
3	32	52.5
Ki-67 labeling index (LI)		
high	36	59.0
low	21	34.4
not tested	4	6.6
Stromal TILs		
LPB	19	31.1
non-LPBC	42	68.9
PD-L1 on stromal TILs (73–10		
positive	37	60.7
negative	24	39.3
Adjuvant chemotherapy		
performed	34	55.7
not performed	24	39.3
undetermined	3	4.9

*LPBC* lymphocyte predominant breast cancer

### CAF PD-L1 expression status using different antibodies

**Fig. 1:**
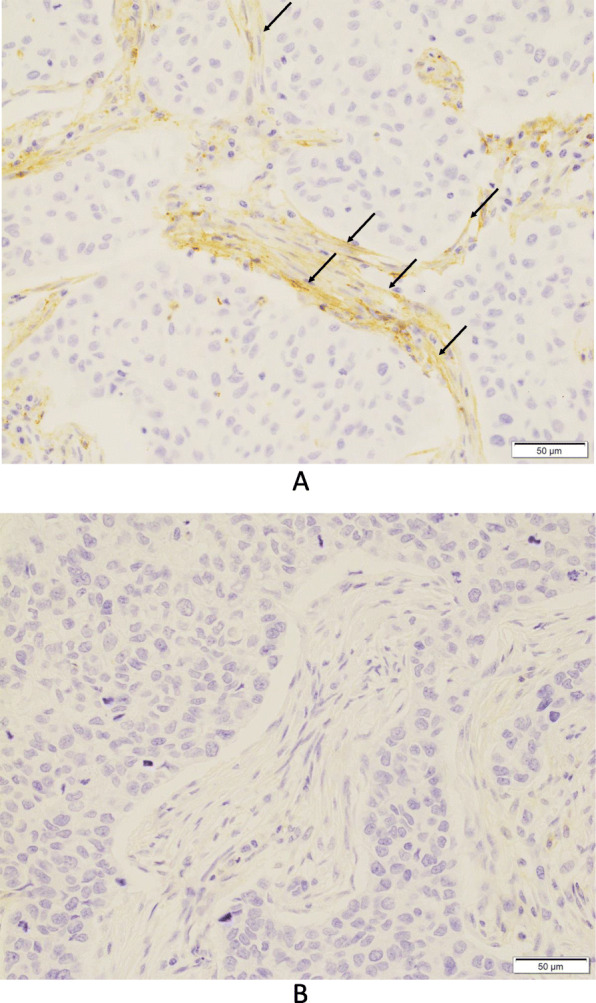
Immunohistochemical staining for programmed death-ligand 1 (PD-L1) (73–10) expression on cancer-associated fibroblasts (CAFs) in triple-negative breast cancer. **a** PD-L1-positive CAFs (arrows). No PD-L1 expression is noted in the carcinoma cells (× 400). **b** PD-L1-negative CAFs (×400)

**Fig. 2:**
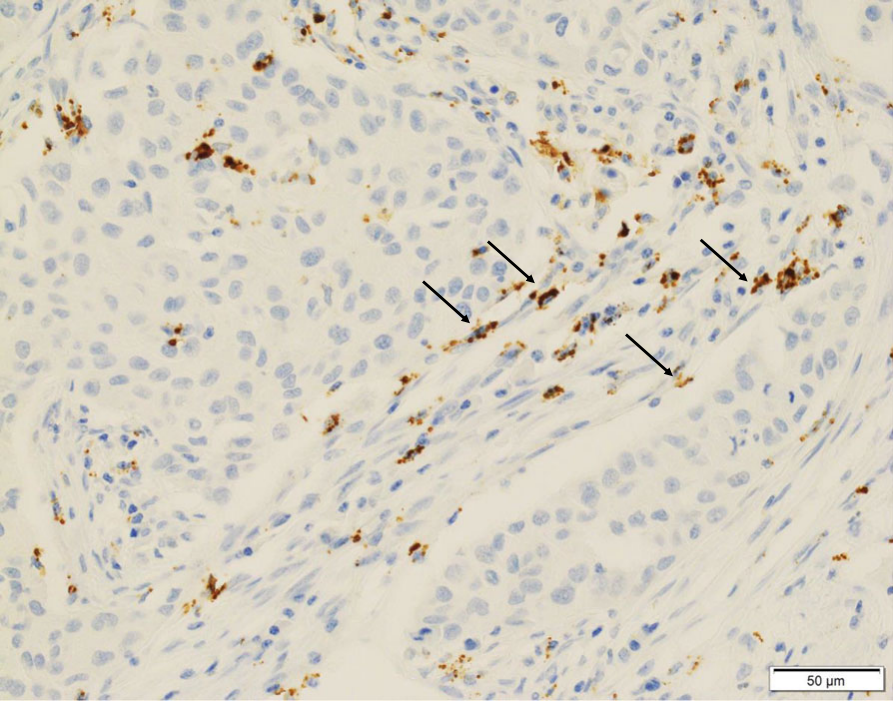
Immunohistochemical staining for PD-L1 (SP-142) expression on cancer-associated fibroblasts (CAFs) in triple-negative breast cancer. PD-L1-positive CAFs (arrows). No PD-L1 expression is noted in the carcinoma cells (× 400)

**Fig. 3:**
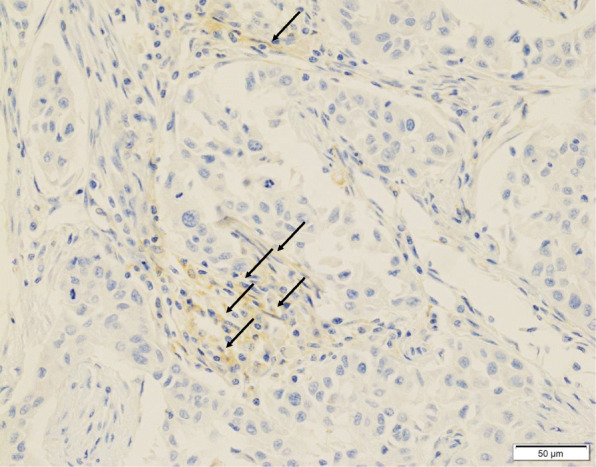
Immunohistochemical staining for PD-L1 (E1L3N) expression on cancer-associated fibroblasts (CAFs) in triple-negative breast cancer. PD-L1-positive CAFs (arrows). No PD-L1 expression is noted in the carcinoma cells (× 400)

In the 73–10 assay, 34 patients (55.7%) were classified as CAF PD-L1-positive (Fig. 1a), and the remaining 27 patients (44.3%) were CAF PD-L1-negative (Fig. 1b). In the SP142 assay, 16 patients (26.2%) were CAF PD-L1-positive (Fig. 2), and the remaining 45 patients (73.8%) were CAF PD-L1-negative. In the E1L3N assay, 25 patients (41.0%) were CAF PD-L1-positive (Fig. 3), and the remaining 36 patients (59.0%) were CAF PD-L1-negative.

### Double immunofluorescence staining

**Fig. 4:**
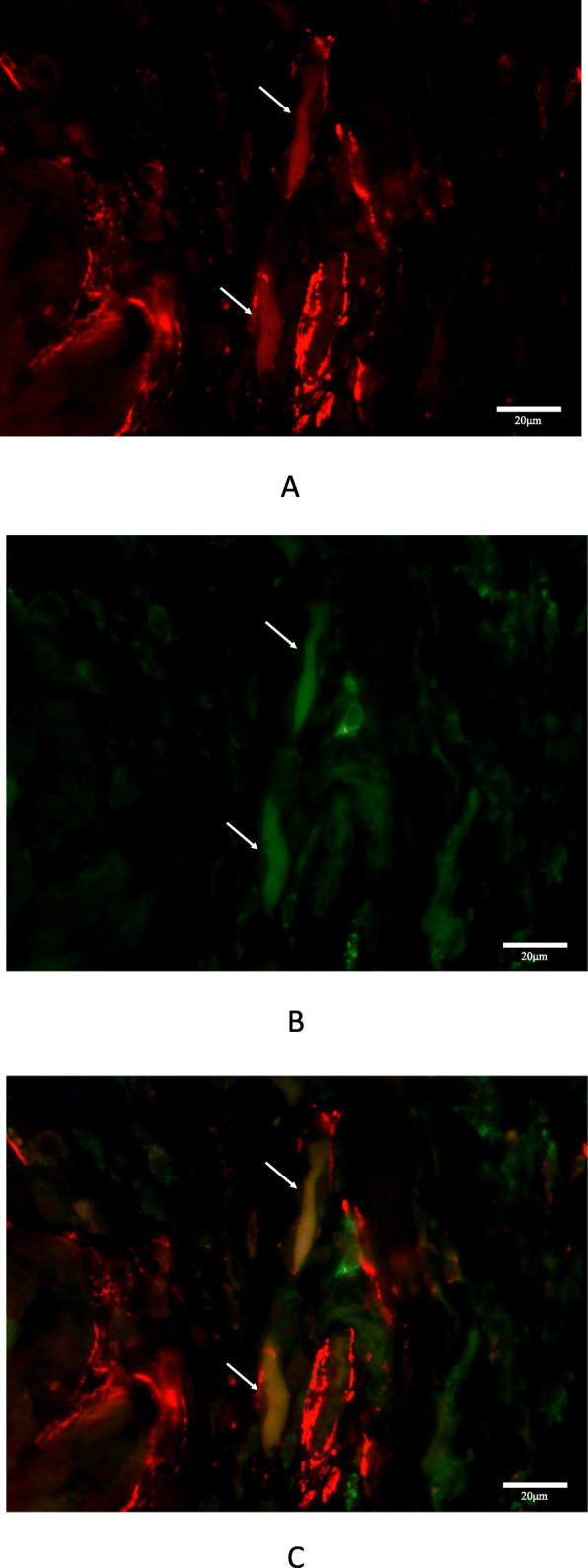
Double immunofluorescence staining in triple-negative breast cancer. **a** α-smooth muscle actin-positive spindle cells stained in red (arrows). **b** PD-L1-positive spindle cells stained in green (arrows). **c** Two merged images of CAFs showing co-expression of α-smooth muscle actin and PD-L1 visualised in yellow (arrows) (× 400)

Immunofluorescence staining revealed spindle-shaped cells around the tumour cells, which co-expressed α-SMA and PD-L1 (Fig. 4a-c). These cells were recognised as CAFs.

### Correlation between CAF PD-L1 expression and clinicopathological factors

Table 2 shows the correlation between CAF PD-L1 expression (73–10) and clinicopathological factors. CAF PD-L1 expression did not correlate with any clinical factors, including age, menopausal status, or presence of adjuvant chemotherapy. Only TIL PD-L1 expression was significantly correlated with CAF PD-L1 expression (*p* < 0.001). CAF PD-L1 expression according to the SP-142 and E1L3N assays was also significantly associated with TIL PD-L1 expression (*p <* 0.001 for both), similar to the 73–10 assay (Tables 3, 4).

**Table 2:** Correlation between clinicopathological factors and CAF PD-L1 expression (73–10 assay)

Factors	PD-L1-positive (*n* = 34)	PD-L1-negative (*n* = 27)	*p*-value
Age (years old; median ± SD)			
	66 ± 13	65 ± 17	0.907
Menopausal status			
premenopausal	4	5	0.482
postmenopausal	30	21	
unknown	0	1	
Tumour size (mm)			
≤ 20	17	14	1.000
> 20	17	13	
Pathological stage			
I + II	30	23	1.000
III	4	4	
Lymph node status			
positive	8	6	0.741
negative	22	11	
not tested	4	10	
Lymphatic invasion			
positive	30	22	0.492
negative	4	5	
Venous invasion			
positive	19	18	0.439
negative	15	9	
Nottingham histlogical grade			
1 + 2	17	12	0.797
3	17	15	
Ki-67 labeling index (LI)			
high	18	10	0.424
low	15	14	
not tested	1	3	
Stromal TILs			
LPB	13	6	0.266
non-LPBC	21	21	
PD-L1 on stromal TILs (73–10)			
positive	29	8	***< 0.001***
negative	5	19	
Adjuvant chemotherapy			
performed	18	16	1.000
not performed	13	11	
undetermined	3	0	

*LPBC* lymphocyte predominant breast cancer

**Table 3:** Correlation between clinicopathological factors and CAF PD-L1 expression (SP142 Assay)

Factors	PD-L1-positive (*n* = 16)	PD-L1-negative (*n* = 45)	*p*-value
Age (years old; median ± SD)			
	68 ± 10	64 ± 16	0.465
Menopausal status			
premenopausal	0	9	0.096
postmenopausal	16	35	
unknown	0	1	
Tumour size (mm)			
≤ 20	7	24	0.570
> 20	9	21	
Pathological stage			
I + II	13	40	0.422
III	3	5	
Lymph node status			
positive	3	11	0.321
negative	13	20	
not tested	0	14	
Lymphatic invasion			
positive	15	37	0.423
negative	1	8	
Venous invasion			
positive	11	26	0.557
negative	5	19	
Nottingham histlogical grade			
1 + 2	6	23	0.395
3	10	22	
Ki-67 labeling index (LI)			
high	9	19	0.749
low	7	22	
not tested	0	4	
Stromal TILs			
LPB	9	10	***0.025***
non-LPBC	7	35	
PD-L1 on stromal TILs (SP142)			
positive	13	12	***< 0.001***
negative	3	32	
not tested	0	1	
Adjuvant chemotherapy			
performed	7	27	0.539
not performed	7	17	
undetermined	2	1	

*LPBC* lymphocyte predominant breast cancer

**Table 4:** Correlation between clinicopathological factors and CAF PD-L1 expression (E1L3N Assay)

Factors	PD-L1-positive (*n* = 25)	PD-L1-negative (*n* = 36)	*p*-value
Age (years old; median ± SD)			
	68 ± 11	64 ± 17	0.268
Menopausal status			
premenopausal	1	8	0.067
postmenopausal	24	27	
unknown	0	1	
Tumour size (mm)			
≤ 20	11	20	0.440
> 20	14	16	
Pathological stage			
I + II	21	32	0.706
III	4	4	
Lymph node status			
positive	6	8	0.534
negative	18	15	
not tested	1	13	
Lymphatic invasion			
positive	22	30	0.725
negative	3	6	
Venous invasion			
positive	15	22	1.000
negative	10	14	
Nottingham histlogical grade			
1 + 2	11	18	0.795
3	14	18	
Ki-67 labeling index (LI)			
high	13	15	0.792
low	12	17	
not tested	0	4	
Stromal TILs			
LPBC	12	7	***0.025***
non-LPBC	13	29	
PD-L1 on stromal TILs (E1L3N)			
positive	12	3	***< 0.001***
negative	13	32	
not tested	0	1	
Adjuvant chemotherapy			
performed	12	22	0.784
not performed	10	14	
undetermined	3	0	

*LPBC* lymphocyte predominant breast cancer

### Correlation between CAF PD-L1 expression and postoperative RFS

The median RFS in CAF PD-L1-positive patients evaluated with 73–10, SP-142, and E1L3N was 59, 61, and 61 months, respectively. The median RFS in CAF PD-L1-negative patients evaluated with 73–10, SP-142, and E1L3N was 47, 53, and 49 months, respectively. CAF PD-L1 expression, evaluated with 73–10, SP-142, and E1L3N did not correlate with RFS (*p* = 0.058, 0.788, and 0.411, respectively).

### Correlation between CAF PD-L1 expression and postoperative OS

**Fig. 5:**
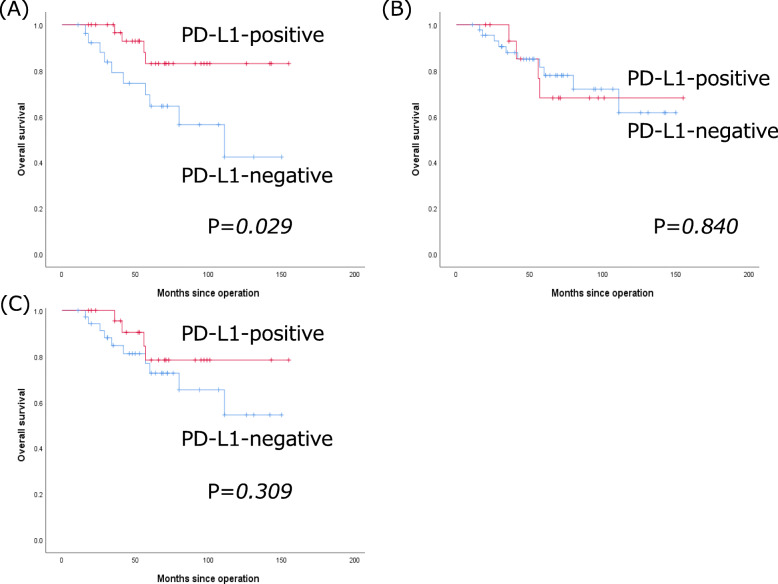
Kaplan–Meier curves for the overall survival (OS) of patients with triple-negative breast cancer. **a** OS curves in cancer-associated fibroblast (CAF) PD-L1-positive (red line) and -negative (blue line) patients evaluated using the 73–10 assay. **b** OS curves in CAF PD-L1-positive (red line) and -negative (blue line) patients evaluated using the SP142 assay. **c** OS curves in CAF PD-L1-positive (red line) and -negative (blue line) patients evaluated using the E1L3N assay

Figure 5 shows the OS curves of CAF PD-L1-positive and -negative patients evaluated using 73–10 (Fig. 5a), SP-142 (Fig. 5b), and E1L3N (Fig. 5c), respectively. The median OS of CAF PD-L1-positive patients evaluated with 73–10, SP-142, and E1L3N was 59, 61, and 61 months, respectively. The median OS in CAF PD-L1-negative patients evaluated with 73–10, SP-142, and E1L3N were 60, 60, and 59 months, respectively. A CAF PD-L1-positive status after evaluation with 73–10 significantly correlated with better OS (*p* = 0.029) in TNBC patients (Fig. 5a). However, CAF PD-L1 expression, evaluated with SP-142 or E1L3N did not correlate with OS (*p* = 0.840 and *p* = 0.309, respectively) (Fig. 5b and c).

### Prognostic significance of CAF PD-L1 expression (73–10)

Based on the univariate analysis, presence of lymph node metastasis and no adjuvant chemotherapy correlated with a poor OS (*p* = 0.001 and 0.016, respectively), and CAF PD-L1 expression significantly correlated with a better OS (*p* = 0.040) (Table 5). Multivariate Cox proportional hazards analyses revealed that CAF PD-L1 expression was an independent factor for a better prognosis of patients with TNBC (hazard ratio [HR]: 0.198; 95% confidence interval [CI]: 0.044–0.891; *p* = 0.035) (Table 5). Moreover, presence of lymph node metastasis and no adjuvant chemotherapy were found to be independent negative prognostic factors for OS (HR: 12.56, 95% CI: 2.465–63.99, *p* = 0.002, and HR: 20.27, 95% CI: 3.041–153.1, *p =* 0.002, respectively).

**Table 5:** Univariate and multivariate analyses for overall survival of patients with triple-negative breast cancer

Factor	HR	Univariate analysis	*P*-value	HR	Multivariate analysis	*P*-value
		95% CI			95% CI	
Tumor size (mm)						
20 < vs ≦ 20	1.801	0.599–5.419	0.295			
Lymph node status						
positive vs negative	9.412	2.465–35.94	***0.001***	12.56	2.465–63.99	***0.002***
Lymphatic invasion						
positive vs negative	1.206	0.155–9.402	0.858			
Venous invasion						
positive vs negative	3.294	0.733–14.80	0.120			
Nottingham histological grade						
3 vs 1 + 2	2.015	0.672–6.035	0.211			
Ki-67 labeling index (LI)						
high vs low	1.199	0.391–3.676	0.750			
Stromal TILs						
LPBC vs. non-LPBC	0.300	0.067–1.352	0.117			
PD-L1 expression on stromal TILs						
positive vs. negative	0.547	0.190–1.579	0.265			
Adjuvant chemotherapy						
not perform vs perform	4.276	1.307–13.99	***0.016***	20.27	3.041–153.1	***0.002***
PD-L1 expression on CAFs						
positive vs. negative	0.297	0.093–0.948	***0.040***	0.198	0.044–0.891	***0.035***

## Discussion

Recent studies on various types of carcinomas have highlighted the important roles of the tumour microenvironment components, including TILs, in cancer growth, invasion, metastasis, and therapeutic resistance [13]. CAFs are some of the dominant components of the tumour microenvironment [13]. CAFs have also been increasing interests in several types of carcinomas, such as head and neck, lung, and rectal carcinomas, because they have been considered to have essential functions in cancer growth and prognosis [18, 27–29]. Recently, PD-L1 expression in CAFs reportedly demonstrated a significantly better prognostic value in patients with non-small cell lung carcinoma [18]. However, the significance of CAFs in breast cancer has not received enough attention.

In the present study, we examined the clinicopathological significance of CAF PD-L1 expression in patients with TNBC and had two main findings: (1) CAF PD-L1 expression was an independent prognostic factor in patients with TNBC and (2) the 73–10 PD-L1 assay would be more suitable for evaluating CAF PD-L1 expression in TNBC, compared to the other two assays. The histopathological grade, lymph node status, tumour size, and Ki-67 LI have been identified as the prognostic factors in patients with TNBC [30]. Furthermore, a recent study revealed that upregulation of PD-L1 was correlated with a good prognosis in patients with TNBC [12]. In our study, a multivariate analysis of OS showed that lymph node status and adjuvant chemotherapy were significant prognostic factors, but histological grade and Ki-67 LI were not. Notably, CAF PD-L1 expression was also established as a significantly better prognostic factor (*p* = 0.035). These results indicate that CAF PD-L1 expression is a novel and useful prognostic factor for OS in patients with TNBC.

It is well known that high PD-L1 expression in the tumour microenvironment is a poor prognostic factor [31]. Stromal PD-L1 was also reported to inhibit the immune responses of CD8-positive T lymphocytes in colorectal cancer [32]. In addition, the expression of PD-L1 on CD8-positive T-cells was a poor prognostic factor in patients with TNBC [31]. These findings suggest that high PD-L1 expression on CAFs could also suppress anti-tumour immune responses through the exhaustion of PD-1-positive lymphocytes. However, in our study, high CAF PD-L1 expression significantly correlated with better prognosis. Moreover, a recent study demonstrated that patients with PD-L1 expression on CAFs had a significantly better prognosis in non-small cell lung cancer, similar to the results of the present cohort analysis. It was also shown that interferon gamma (IFN-γ) activated PD-L1 expression on CAFs [18]. PD-L1 on CAFs was upregulated through interaction with IFN-γ, hence releasing activated lymphocytes; furthermore, PD-L1 expression on CAFs indicated abundant infiltration of TILs in the tumour microenvironment [18]. Nevertheless, there was no significant association between CAF PD-L1 expression and TILs in non-small cell lung cancer [18] and TNBC assessed with the 73–10 assay in our study (stromal TILs were significantly associated with CAF PD-L1 positivity when assessed using SP142 and E1L3N assays in the present cohort). Thus, immune cells other than TILs such as macrophages might have played a role in this discrepancy for specific sources of IFN-γ. Further studies are hence needed to clarify the underlying molecular mechanism.

Several immunohistochemical assays for PD-L1 have been independently developed for companion diagnostics to determine the indication for anti-PD-L1/PD1 targeted therapy. Interestingly, only CAF PD-L1 expression determined using the 73–10 assay was significantly correlated with better OS (results of the SP-142 and E1L3N assays did not) in our cohort. The 73–10 antibody is known to bind to the region of the C-terminal cytoplasmic domain of PD-L1 [33]. Although SP-142 and E1L3N are also known to bind to the C-terminal cytoplasmic domain of PD-L1, it has been reported that they have slightly different binding sites [34, 35]. SP142 binds to an epitope in the cytoplasmic domain at the extreme C-terminus, and several mutations lead to lack of immunostaining for SP142 [35]. Although the specific binding site of 73–10 has not been reported, it is speculated that 73–10 binds to a different intracytoplasmic domain of PD-L1, from those of SP142 and E1L3N, resulting in the difference in staining properties in non-small cell lung cancer [33, 35, 36]. CAF PD-L1 expression was detected the most by the 73–10 assay, compared to the SP142 and E1L3N assays; this might be a reflection of the difference of binding sites among primary antibodies. The 73–10 antibody would therefore be more suitable for studying PD-L1 expression in CAFs of patients with TNBC. Furthermore, in this study, we demonstrated that spindle cells around the tumour cells co-expressed α-SMA and PD-L1 with immunofluorescence staining, and these cells were considered as PD-L1-expressing CAFs, because α-SMA is one of the most common markers of CAFs in breast cancer [14].

Nevertheless, it is important to note that some limitations were present in our study. This was a retrospective study with a small sample size that could have led to selection bias. Because tissue microarrays were used to determine CAF PD-L1 expression, cancer tissue may have shown heterogeneous expression, despite that we have selected the morphologically most representative regions of the cancer tissue. Finally, this study was focussed on the expression of CAF PD-L1 in TNBC. PD-L1 expression in stromal cells differs among molecular subtypes of breast cancer [37], hence CAF PD-L1 expression might also be different in luminal and HER2 subtypes. Further analyses are needed to clarify the prognostic value of CAF PD-L1 expression in patients with breast cancer subtypes other than the TNBC.

## Conclusions

This study demonstrates that CAF PD-L1 expression is an independent better prognostic factor in patients with TNBC, which could have implications in diagnosis, disease management, and the development of targeted therapeutics. Nevertheless, additional studies are needed to elucidate the molecular mechanisms involved in CAF PD-L1 expression in TNBC and to develop therapeutic interventions for patients with CAF PD-L1-positive TNBC. Moreover, the 73–10 assay may be the most suitable for immunostaining of CAF PD-L1 in TNBC.

## Data Availability

The datasets used and/or analysed during the current study are available from the corresponding author on reasonable request.

## Abbreviations

CAFs: Cancer-associated fibroblasts
HER2: Human epidermal growth factor receptor 2
IFN-γ: Interferon gamma
Ki-67 LI: Ki-67 labelling index
OS: Overall survival
PD-L1: Programmed death ligand 1
RFS: Relapse-free survival
TNBC: Triple-negative breast cancer
TILs: Tumour-infiltrating lymphocytes
